# Intracellular ion channels and cancer

**DOI:** 10.3389/fphys.2013.00227

**Published:** 2013-09-03

**Authors:** Luigi Leanza, Lucia Biasutto, Antonella Managò, Erich Gulbins, Mario Zoratti, Ildikò Szabò

**Affiliations:** ^1^Department of Biology, University of PadovaPadova, Italy; ^2^CNR Institute of Neuroscience and Department of Biomedical Sciences of the University of PadovaPadova, Italy; ^3^Department of Molecular Biology, University of Duisburg-EssenEssen, Germany

**Keywords:** cancer, organelles, ion channel, apoptosis, pharmacology

## Abstract

Several types of channels play a role in the maintenance of ion homeostasis in subcellular organelles including endoplasmatic reticulum, nucleus, lysosome, endosome, and mitochondria. Here we give a brief overview of the contribution of various mitochondrial and other organellar channels to cancer cell proliferation or death. Much attention is focused on channels involved in intracellular calcium signaling and on ion fluxes in the ATP-producing organelle mitochondria. Mitochondrial K^+^ channels (Ca^2+^-dependent BK_Ca_ and IK_Ca_, ATP-dependent K_ATP_, Kv1.3, two-pore TWIK-related Acid-Sensitive K^+^ channel-3 (TASK-3)), Ca^2+^ uniporter MCU, Mg^2+^-permeable Mrs2, anion channels (voltage-dependent chloride channel VDAC, intracellular chloride channel CLIC) and the Permeability Transition Pore (MPTP) contribute importantly to the regulation of function in this organelle. Since mitochondria play a central role in apoptosis, modulation of their ion channels by pharmacological means may lead to death of cancer cells. The nuclear potassium channel Kv10.1 and the nuclear chloride channel CLIC4 as well as the endoplasmatic reticulum (ER)-located inositol 1,4,5-trisphosphate (IP_3_) receptor, the ER-located Ca^2+^ depletion sensor STIM1 (stromal interaction molecule 1), a component of the store-operated Ca^2+^ channel and the ER-resident TRPM8 are also mentioned. Furthermore, pharmacological tools affecting organellar channels and modulating cancer cell survival are discussed. The channels described in this review are summarized on Figure 1. Overall, the view is emerging that intracellular ion channels may represent a promising target for cancer treatment.

## Mitochondria

The “impermeable” mitochondrial inner membrane (IMM) allows the formation of an electrochemical proton gradient which drives the aerobic synthesis of ATP. The “semipermeable” outer membrane (OMM) encloses a periplasmic space where proteins with fundamental roles in cell death are stored until a sufficiently strong pro-apoptotic signal arrives. Mitochondria have assumed a peculiar role in cancer cell physiology (Ralph and Neuzil, 2009). They are crucial for the control of intracellular Ca^2+^ homeostasis, and produce reactive oxygen species (ROS). ROS are involved in the regulation of physiological processes, but may also be harmful if produced excessively. Mitochondria are the checkpoint of the intrinsic pathway of apoptosis: the release of caspase cofactors, such as cytochrome c (cyt c) and SMAC/Diablo, results in the assembly of the apoptosome and in commitment of the cell to apoptosis. In cancer cells mitochondrial metabolism is deregulated to optimize the production of glycolytic intermediates for anabolic reactions. Much effort has been devoted to discover drugs inducing cancer cell death by targeting tumor-specific alterations of mitochondrial metabolism or by stimulating OMM permeabilization and thus, allowing the release of apoptotic co-factors (Fulda et al., 2010). Mitochondrial ion channels play a role in this process by influencing organellar membrane potential, ROS production, volume, calcium homeostasis, and possibly morphology. The mitochondrial channels characterized over the last two decades include outer membrane-located VDAC and MAC in the IMM, potassium channels mtK_ATP_, mtBK_Ca_, mtIK_Ca_, mtKv1.3, TASK-3, the non-selective permeability transition pore PTP, chloride channels, and the calcium uniporter (e.g., Zoratti et al., 2009; Shoshan-Barmatz et al., 2010; Rizzuto et al., 2012; Szabò et al., 2012).

**Figure 1:**
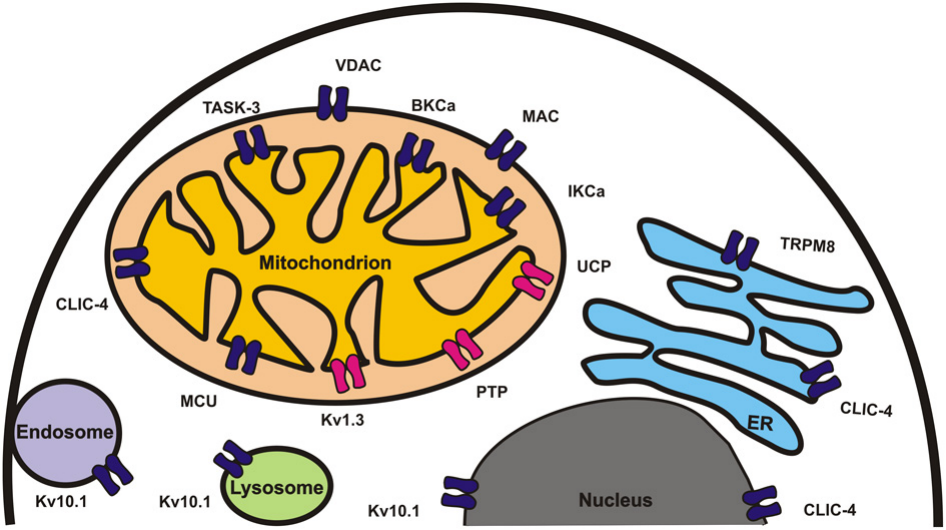
**Ion channels involved in regulation of apoptosis and/or tumorigenesis are shown in different organelles.** Channels for whose crucial role pharmacological and/or genetic *in vivo* evidence is available are shown in red.

### Channels of the outer mitochondrial membrane involved in apoptosis/cancer

#### Mitochondrial apoptosis-induced channel (MAC)

OMM permeabilization has been proposed to involve oligomers of pro-apoptotic Bax, which display ion channel activity in phospholipid bilayers (e.g., Tait and Green, 2010). However, the hypothesis that Bax alone is sufficient to induce cyt c release has been challenged, given that a single point mutant of Bax did not mediate cell death in Bax/Bak-less mouse embryonic fibroblasts despite forming channels with properties similar to WT Bax (Brustovetsky et al., 2010; Szabò et al., 2011). A pore (mitochondrial apoptosis-induced channel, MAC) with an estimated diameter sufficient to allow the passage of cyt c was detected by patch clamp (Martinez-Caballero et al., 2009). The timing of cyt c release in apoptotic cells correlated with the onset of MAC activity and with the translocation of Bax to mitochondrial membranes. MAC, whose formation requires Bim-induced activation of Bax and a still unidentified protein, is considered as a target for novel cancer therapies (Peixoto et al., 2012) but specific MAC activators are not available yet. The BH3 mimetic ABT-737, an efficient anti-cancer agent *in vivo*, activates MAC by disrupting Bcl-2/Bax/Bim complexes (Dejean et al., 2010).

#### Mitochondrial voltage dependent anion channel (VDAC)

The major protein of the OMM, porin or VDAC is deeply involved in apoptosis. The role of VDAC1 and of the other isoforms VDAC2 and VDAC3 in cell death is multi-faceted and complex (e.g., McCommis and Baines, 2012; Shoshan-Barmatz and Golan, 2012; Shoshan-Barmatz and Mizrachi, 2012). Formation of a large pore comprising VDAC and Bax/Bak was proposed to account for cyt c release (Tsujimoto and Shimizu, 2000; but see Martinez-Caballero et al., 2009). Alternatively, dimers and higher oligomers of VDAC1 might form the conduit for the efflux of cyt c (Shoshan-Barmatz et al., 2010). Binding of anti-apoptotic Bcl-2 and BclxL to VDAC1 (with resulting inhibition of porin) (Shimizu et al., 2000) has an anti-apoptotic action (e.g., Arbel et al., 2012). In contrast, block of VDAC1 by the phosphorothioate oligonucleotide G3139 (Tan, 2012) or by avicins (plant saponins with anticancer activity) is pro-apoptotic, presumably by reducing flux of metabolites across the OMM (Haridas et al., 2007). VDAC2 inhibits Bak activation and apoptosis (Cheng et al., 2003), and Bak reportedly relocates from the OMM to the ER in the absence of VDAC2 (Raghavan et al., 2012). In contrast, Bax-induced cyt c release from mitochondria isolated from WT or VDAC1^−^, VDAC3^−^ and VDAC1/VDAC3-null cells was reported to be the same (Baines et al., 2007).

VDAC may inhibit apoptosis and promote tumorigenesis through specific interactions with enzymes favoring glycolysis. It is being examined as a cancer-specific target since tumor cells have elevated glycolysis and increased expression of VDACs (Grills et al., 2011). Overexpression of Hexokinase-2 (HK2) and its association with VDAC are key features of glycolytic cancers (e.g., Wolf et al., 2011). HK2 binding to the conduit channeling ATP out of mitochondria provides a metabolic benefit to cancer cells (Warburg effect) and it antagonizes cell death via inhibition of Bax-induced cyt c release (Pastorino et al., 2002; Gall et al., 2011) and/or inhibition of the Mitochondrial Permeability Transition (MPT) (Chiara et al., 2008). HK detachment seems to favor cell death by disruption of aerobic glycolysis and of the energy balance of the cell, regulation of ROS production, altered interaction of Bcl2 family proteins with mitochondria, facilitation of VDAC oligomer formation (e.g., Shoshan-Barmatz et al., 2010; Shoshan-Barmatz and Golan, 2012). Therefore, a major oncological target is the HK-VDAC complex (e.g., Galluzzi et al., 2008; Simamura et al., 2008; Fulda et al., 2010; Mathupala and Pedersen, 2010). HK2 can be dissociated from mitochondria by peptides interfering with HK-VDAC association, by erastin (Yagoda et al., 2007) and by 3-bromopyruvate (e.g., Cardaci et al., 2012; Ko et al., 2012; Pedersen, 2012; Shoshan, 2012). Antifungal drugs clotrimazole and bifonazole and the plant hormone methyl jasmonate (MJ) are also effective. MJ is particularly promising since it has selective anticancer activity in preclinical studies (Fulda et al., 2010). Finally, the anti-cancer agent furanonaphthoquinone (FNQ) induces caspase-dependent apoptosis via the production of ROS, which is enhanced by VDAC1 overexpression (Simamura et al., 2008). A systematic search for compounds acting at the level of VDAC to antagonize cancer remains to be performed.

### Ion channels of the inner mitochondrial membrane involved in apoptosis/cancer

#### Permeability transition pore (MPTP)

When the IMM becomes freely permeable to solutes, the consequences for the cell can be catastrophic. Thus, the selective induction of IMM permeabilization in cancer cells is a strategy worth pursuing in oncotherapy. A number of cellular stresses and cytotoxic agents trigger the prime example of such a catastrophe, i.e., the mitochondrial permeability transition (MPT), considered as a final common pathway of cell death (Brenner and Grimm, 2006; Bernardi, 2013). The MPT is caused by the opening of a large Ca^2+^- and oxidative stress-activated pore [the mitochondrial megachannel, MMC, with a conductance of up to 1.5 nS (Szabó and Zoratti, 1991)] which makes the IMM permeable to ions and solutes up to about 1500 Da MW, leading to matrix swelling.

MPT is considered to bear substantial responsibilities in the tissue damage caused by, e.g., ischemia/reperfusion and oxidative stress. In cancer cells, instead, signaling pathways are activated which desensitize the mitochondria to MPT induction (Rasola et al., 2010; Matassa et al., 2012; Traba et al., 2012), while chemotherapeutic agents causing oxidative stress may activate signals causing death via the MPT (Chiara et al., 2012). Cyclosporin A (CSA), a cyclic endecapeptide, is a powerful inhibitor of the MPTP (Fournier et al., 1987; Crompton et al., 1988; Broekemeier et al., 1989) (and also of calcineurin and thus is a widely used immunosuppressant). CSA inhibits the MPTP via its binding to matrix cyclophilin (CyP) D, a peptidyl-prolyl cis-trans isomerase (PPIase). Patients treated with CSA to prevent transplant rejection have a high incidence of cancer not only because of the drug's immunosuppressive action, but also because CSA inhibits the MPTP (Norman et al., 2010). The molecular nature of the MPTP is being finally delineated: the dimeric form of ATP synthase and CypD as regulator are currently proposed as components (Baines et al., 2005; Bernardi, 2013; Giorgio et al., 2013).

For oncological applications MPT inducers are relevant, despite the likelihood of noxious side-effects, for example on the nervous system. A large number of compounds, often used at relatively high concentrations, have been shown to induce the MPT in cultured cells, often as a consequence of oxidative stress and/or disruption of Ca^2+^ homeostasis. Some MPTP-targeting molecules such as 4-(N-(S-glutathionylacetyl) amino) phenylarsenoxide are currently being evaluated in clinical trials for cancer treatment of refractory tumors (Brenner and Moulin, 2012; Elliott et al., 2012).

Signaling pathways which modulate occurrence of the MPT have been elucidated, a key component being GSK3α/β whose activation, e.g., by induction of oxidative stress by gold complex AUL12, favors MPTP opening (Chiara and Rasola, 2013). A large portion of MPTP openers are natural compounds like jasmonates (e.g., Raviv et al., 2013), betulinic acid, the synthetic retinoid CD437 (Lena et al., 2009; Javadov et al., 2011), berberine (Pereira et al., 2007, 2008), honokiol (Li et al., 2007), α-bisabolol (Cavalieri et al., 2009) and shikonin (Han et al., 2007), just to name a few. Data on *in vivo* anti-tumor activities are available for all these compounds (Fulda et al., 2010). Mitochondria-penetrating peptides, such as mastoparan-like sequences, peptides of the innate immunity systems, or the molecules developed by Kelley's group (e.g., Risso et al., 2002; Jones et al., 2008; Horton et al., 2012) also induce MPT. Some MPTP-targeting molecules such as 4-(N-(S-glutathionylacetyl) amino) phenylarsenoxide are currently being evaluated in clinical trials for cancer treatment of refractory tumors (Brenner and Moulin, 2012; Elliott et al., 2012).

#### IMM potassium channels Kv1.3, BKca, IKca, and TASK-3 in the regulation of apoptosis/cancer

A functional mitochondrial counterpart of the potassium channel Kv1.3 has been identified in the IMM of several cell types (mtKv1.3) (Szabò et al., 2005; Gulbins et al., 2010). It is expected to participate in regulation of mitochondrial membrane potential, volume, and ROS production. A crucial role of mtKv1.3 in apoptosis became evident since expression of a mitochondria-targeted Kv1.3 construct was sufficient to sensitize apoptosis-resistant CTLL-2 T lymphocytes, which lack Kv channels. MtKv1.3 has been identified as a target of Bax and physical interaction between the two proteins in apoptotic cells has been demonstrated (Szabó et al., 2008; Szabò et al., 2011). Incubating Kv1.3-positive isolated mitochondria with Bax triggered apoptotic events including membrane potential changes (hyperpolarization followed by depolarization due to the opening of MPTP), ROS production and cyt c release, whereas Kv1.3-deficient mitochondria were resistant. Highly conserved Bax lysine 128 protrudes into the intermembrane space (Annis et al., 2005) and mimics a crucial lysine in Kv1.3-blocking peptide toxins. Mutation of Bax at K128 (BaxK128E) abrogated its effects on Kv1.3 and mitochondria, as well as in Bax/Bak-less double knock-out (DKO) mouse embryonic fibroblasts, indicating a toxin-like action of Bax on Kv1.3 to trigger mitochondrial phenomena.

Psora-4, PAP-1 and clofazimine, three membrane-permeant inhibitors of Kv1.3, can induce death by directly targeting the mitochondrial channel, while membrane-impermeant Kv1.3 inhibitors ShK or Margatoxin did not induce apoptosis (Leanza et al., 2012a,b). Importantly, the membrane-permeant drugs killed cells also in the absence of Bax and Bak, in agreement with the above model. Genetic deficiency or siRNA-mediated downregulation of Kv1.3 abrogated the effects of the drugs. Intraperitoneal injection of clofazimine reduced tumor size by 90% in an orthotopic melanoma B16F10 mouse model *in vivo*, while no adverse effects were observed in several healthy tissues. Similar results were obtained with primary human cancer cells from patients with chronic lymphocytic leukemia (Leanza et al., 2013). The selective action of these drugs on tumor cells is related to a synergistic effect of a higher expression of Kv1.3 and of an altered redox state of cancer cells. The fact that clofazimine is already used in the clinic for the treatment of e.g., leprosis (Ren et al., 2008) and shows an excellent safety profile supports the feasibility of targeting mtKv1.3 for therapy.

The large conductance calcium- and voltage-activated K^+^ channel BK_Ca_ (KCa1.1) has been revealed also in intracellular membranes, including nuclear membrane, ER, Golgi and mitochondria (Xu et al., 2002; O'Rourke, 2007; Singh et al., 2012). Patch clamp experiments with recombinant Bax showed an inhibition of BK_Ca_, which might contribute to opening of the MPTP during cell death (Cheng et al., 2011).

The intermediate conductance potassium channel (IK_Ca_; KCa3.1), selectively inhibited by clotrimazole and TRAM-34, has been recorded from the inner mitochondrial membranes of human cancer cells (De Marchi et al., 2009; Sassi et al., 2010). TRAM-34 used alone did not induce apoptosis (Sassi et al., 2010; Quast et al., 2012), but it synergistically increased sensitivity to the death receptor ligand TRAIL in melanoma cells (Quast et al., 2012). Given that both TRAM-34 and TRAIL have a relatively good safety profile, co-administration of the two drugs might be exploited for melanoma treatment.

Recently TASK-3 (KCNK9), a two-pore potassium channel, was identified in mitochondria of melanoma and keratinocyte (Rusznák et al., 2008) as well as healthy intestinal epithelial cells (Kovács et al., 2005). Reduced expression of TASK-3 resulted in compromised mitochondrial function and cell survival in WM35 melanoma cells (Kosztka et al., 2011). Whether TASK-3 protein gives rise to a functional channel in the IMM and whether it will become an oncological target remain to be determined.

### Other IMM channels linked to tumorigenesis: uncoupling protein UCP, Mg^2+^ channel Mrs-2 and calcium uniporter MCU

Uncoupling protein-2 (UCP-2), which mediates proton leak (Cannon and Nedergaard, 2004; Fedorenko et al., 2012), has been proposed to regulate cell survival by decreasing mitochondrial ROS, since a depolarizing proton leak is expected to diminish superoxide production (Baffy et al., 2011). UCP2 over-expression reportedly prevents oxidative injury, thereby possibly contributing to a higher apoptotic threshold assisting survival of cancer cells. Over-expression of UCP2 was found in numerous types of tumors and has been shown to protect cells from oxidative stress (Arsenijevic et al., 2000; Zhang et al., 2007) and even to abolish chemotherapeutic agent- induced apoptosis (Derdak et al., 2008). Ectopic expression of UCP2 in MCF7 breast cancer cells leads to a decreased mitochondrial membrane potential and increased tumorigenic properties as measured by cell migration, *in vitro* invasion, and anchorage independent growth. Interestingly, UCP2 over-expression has also been proposed to directly contribute to the Warburg phenotype (Samudio et al., 2008) and to development of tumors in an orthotopic model of breast cancer (Ayyasamy et al., 2011). Cisplatin downregulated the expression of UCP2 in colon cancer cells (Santandreu et al., 2010), suggesting that UCP2 over-expression is involved in the development of a variety of cancers. UCP2 can be considered as a promising oncological target.

Mitochondria accumulate Mg^2+^ via Mrs2, a Mg^2+^–selective channel of the IMM (Kolisek et al., 2003). An early increase in cytosolic Mg^2+^ occurs during apoptosis (Chien et al., 1999) and this ion seems to be required for cytochrome c release (Kim et al., 2000). Long-lasting knock-down of Mrs2 caused cell death by inducing loss of respiratory complex I and mitochondrial membrane depolarization (Piskacek et al., 2009). A subtractive hybridization method applied on vincristine or adriamycin resistant and parental human gastric adenocarcinoma cell lines highlighted upregulation of Mrs2 (Chen et al., 2009), suggesting that high expression of Mrs2 may protect against death (Wolf and Trapani, 2009).

The molecular identification of the mitochondrial Ca^2+^ “uniporter” (MCU), responsible for the low-affinity uptake of calcium into the mitochondrial matrix (Kirichok et al., 2004), has recently been achieved (Baughman et al., 2011; De Stefani et al., 2011). MCU participates in the control of Ca^2+^ signaling in the whole cell, and may thus be a very useful tool to influence the myriad cellular calcium-dependent processes, including cell death (Rizzuto et al., 2012). Subthreshold apoptotic signals were shown to synergize with cytosolic Ca^2+^ waves (Pinton et al., 2001) resulting in opening of MPTP. Cells overexpressing MCU underwent more pronounced apoptosis upon challenging with H_2_O_2_ and C2-ceramide (De Stefani et al., 2011). Overexpression of an MCU-targeting microRNA, miR-25, in colon cancer cells resulted in MCU downregulation, impaired calcium uptake and increased resistance to apoptosis (Marchi et al., 2013). Thus, MCU seems to be a crucial protein for tumorigenesis and its specific pharmacological activators, if identified, might become useful tools.

## Ion channels in other organelles with a role in apoptosis/tumorigenesis

The intracellular chloride channel CLIC4/mtCLIC has both a soluble and a membrane-inserted form and can be localized to the mitochondrial inner membrane (Fernández-Salas et al., 1999), cytoplasm, ER membrane, and the nucleus. CLIC4 overexpression induced apoptosis associated with loss of mitochondrial membrane potential, cytochrome c release, and caspase activation (Fernández-Salas et al., 2002). On the other hand, inhibition of CLIC4 expression triggered mitochondrial apoptosis under starvation and enhanced autophagy in glioma cells (Zhong et al., 2012). Marked changes in expression and subcellular localization of CLIC4 occur early in tumorigenesis. In particular, reduced CLIC4 expression and nuclear localization in cancer cells is associated with the altered redox state and CLIC4 acts as an important suppressor of squamous tumor development and progression (Suh et al., 2012).

A functional “oncogenic” potassium channel, Kv10.1 has been described in the nuclear inner membrane (Chen et al., 2011) where it might participate in setting nuclear [K^+^] thereby affecting gene expression. The PM Kv10.1 is also rapidly internalized to lysosomes (Kohl et al., 2011), whose patch clamping has been achieved (Wang et al., 2012). The possible influence of these channels on cancer cell survival remains to be determined.

Finally, we should briefly mention other intracellular channels involved in Ca^2+^ signaling, (Ca^2+^ permeable channels are discussed in detail by other contributions in this special issue). For example the calcium-permeable ion channel TRPM8, overexpressed in several tumors, has been located to the ER (Zhang and Barritt, 2004), resulting in decreased ER [Ca^2+^] and increased resistance to apoptosis (Bidaux et al., 2007). Patients suffering of breast cancers with high ER-located STIM1 levels have significantly reduced survival (McAndrew et al., 2011). The PM-located other component, ORAi1 contributes to altered calcium homeostasis as well (Monteith et al., 2012). Expression of ER-resident IP_3_ receptors acting as Ca^2+^ store release channels is altered in glioblastoma (Kang et al., 2010). Repression of IP_3_- mediated Ca^2+^ elevation by Bcl-2 has been proposed to contribute to the pathophysiology of chronic lymphocytic leukemia (Zhong et al., 2011).

In summary, while considerable further work is required to clarify the mechanisms by which intracellular channels contribute to tumorigenesis and tumor progression, or intervene in cell death, a few *in vivo* studies targeting these channels underline the importance of pursuing this line of research.

### Conflict of interest statement

The authors declare that the research was conducted in the absence of any commercial or financial relationships that could be construed as a potential conflict of interest.
